# Clinical and mechanistic responses to a music-based intervention for pain in adults with irritable bowel syndrome: Protocol for a single-arm pilot study

**DOI:** 10.64898/2026.09.11.26362754

**Published:** 2026-09-17

**Authors:** Weizi Wu, Eunhea You, Aolan Li, Jie Chen, Luana Colloca, Shannon Kiley, Boluwatife Faremi, Camila Jimenez Wong, John M. Toribio, Kyle J. Mahoney, Hugo Fernando Posada-Quintero, Ming-Hui Chen, Debra S Burns, Xiaomei Cong

## Abstract

Irritable bowel syndrome (IBS) is a common disorder of brain-gut interaction characterized by recurrent abdominal pain and altered bowel habits. Although music-based interventions (MBIs) have shown benefits on pain and stress, the mechanisms associated with MBI-related changes in IBS pain remain poorly understood, and the feasibility of capturing these responses using multimodal wearable monitoring in everyday settings has not been established. This protocol describes a single-arm pilot mechanistic trial examining clinical and multimodal mechanistic responses to an MBI in adults with IBS and the feasibility of extending physiological monitoring from the laboratory to home settings. Thirty-six adults aged 18-50 years with provider-confirmed IBS will be enrolled, with approximately 30 expected to complete the post-intervention laboratory visit. Participants will receive board-certified music therapist-guided laboratory sessions and complete four weeks of self-administered home practice using a standardized 20-minute MBI protocol at least five days per week. Guided by a brain-gut mechanistic framework, assessments will include patient-reported pain, stress, and IBS symptoms; quantitative sensory testing; gut microbiome profiling using 16S rRNA gene sequencing; and multimodal physiological recordings of neural, autonomic, and muscular activity. Analyses will characterize within-session and longitudinal changes in clinical and mechanistic measures and assess recruitment, retention, MBI adherence, wearable data completeness, and participant acceptability. Findings will inform the selection of candidate mechanisms and wearable monitoring approaches for evaluation in a future adequately powered randomized controlled trial.

## Full Text Availability

The license terms selected by the author(s) for this preprint version do not permit archiving in PMC. The full text is available from the preprint server.

